# Efficient differentially private learning improves drug sensitivity prediction

**DOI:** 10.1186/s13062-017-0203-4

**Published:** 2018-02-06

**Authors:** Antti Honkela, Mrinal Das, Arttu Nieminen, Onur Dikmen, Samuel Kaski

**Affiliations:** 1 Helsinki Institute for Information Technology HIIT, Department of Computer Science, University of Helsinki, Helsinki, Finland; 2 Department of Mathematics and Statistics, University of Helsinki, Helsinki, Finland; 3 Department of Public Health, University of Helsinki, Helsinki, Finland; 4 Helsinki Institute for Information Technology HIIT, Department of Computer Science, Aalto University, Helsinki, Finland

**Keywords:** Differential privacy, Linear regression, Drug sensitivity prediction, Machine learning

## Abstract

**Background:**

Users of a personalised recommendation system face a dilemma: recommendations can be improved by learning from data, but only if other users are willing to share their private information. Good personalised predictions are vitally important in precision medicine, but genomic information on which the predictions are based is also particularly sensitive, as it directly identifies the patients and hence cannot easily be anonymised. Differential privacy has emerged as a potentially promising solution: privacy is considered sufficient if presence of individual patients cannot be distinguished. However, differentially private learning with current methods does not improve predictions with feasible data sizes and dimensionalities.

**Results:**

We show that useful predictors can be learned under powerful differential privacy guarantees, and even from moderately-sized data sets, by demonstrating significant improvements in the accuracy of private drug sensitivity prediction with a new robust private regression method. Our method matches the predictive accuracy of the state-of-the-art non-private lasso regression using only 4x more samples under relatively strong differential privacy guarantees. Good performance with limited data is achieved by limiting the sharing of private information by decreasing the dimensionality and by projecting outliers to fit tighter bounds, therefore needing to add less noise for equal privacy.

**Conclusions:**

The proposed differentially private regression method combines theoretical appeal and asymptotic efficiency with good prediction accuracy even with moderate-sized data. As already the simple-to-implement method shows promise on the challenging genomic data, we anticipate rapid progress towards practical applications in many fields.

**Reviewers:**

This article was reviewed by Zoltan Gaspari and David Kreil.

**Electronic supplementary material:**

The online version of this article (10.1186/s13062-017-0203-4) contains supplementary material, which is available to authorized users.

## Background

The widespread collection of private data, in the health domain both by individuals and hospitals, creates a major opportunity to develop new services by learning predictive models from the data. Privacy-preserving algorithms are required and have been proposed, but for instance anonymisation approaches [1–3] cannot guarantee privacy against adversaries with additional side information, and are poorly suited for genomic data where the entire data vector is identifying [4]. Guarantees of differential privacy [5, 6] remain valid even under these conditions [6], and differential privacy has arisen as the most popularly studied strong privacy mechanism for learning from data.

Genomics is an important domain for privacy-aware modelling, in particular for precision medicine. Many people wish to keep their and also their relatives’ genomes private [7], and simple anonymisation is not sufficient to protect the privacy since a genome is inherently identifiable [4]. Furthermore, individual genomes can be recovered from summary statistics [8] as well as phenotype data such as gene expression data [9]. Hence, the hospital or clinic holding the genomic data will need to be very cautious about privacy risks when releasing any genomic data, even though the data would be needed and useful for future diagnoses and treatment decisions. These findings have motivated a number of privacy-preserving methods for genome-wide association studies, based on differential privacy [10–12] as well as relaxations that provide more accurate modelling results under weaker privacy guarantees [13]. Previous research in drug dosing for personalised medicine has shown that inefficient differentially private models may put the patients at severe risk [14].

**Fig. 1:**
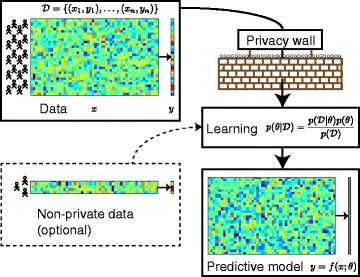
Typical modelling setup for differentially private learning of a predictive model. In many applications most data (top) are available for learning only if their privacy can be protected

Our work for this paper is motivated by modelling for personalised medicine. One possible learning scenario in this field is illustrated in Fig. 1 where the party developing the predictive model has unrestricted access to at most a very limited data set (bottom left), for example from local patients willing to share their data or from large public research projects with liberal data sharing practices. At the same time there are potentially a lot more data available from other sources (top), but access to those is constrained by privacy concerns. A similar setting was considered previously in [15], which presents a simple mechanism for combining public and private data for logistic regression, but the results they obtain are quite inaccurate. In contrast, our approach for linear regression is asymptotically efficient and yields significantly more accurate results for reasonably-sized privacy-protected data sets than any previous method. This creates a promise for new type of data sharing that can find effective compromises between the utility of the data for learning new models and the privacy of the data subjects.

## Approach

### Efficient differentially private learning

Differential privacy [5, 6] is a formulation of reasonable privacy guarantees for privacy-preserving computation. It gives guarantees about the output of a computation and can be combined with complementary cryptographic approaches such as homomorphic encryption [16] if the computation process needs protection too. An algorithm $\mathcal {M}$ operating on a data set $\mathcal {D}$ is said to be *differentially private* if for any two data sets $\mathcal {D}$ and $\mathcal {D}'$, differing only by one sample, the ratio of probabilities of obtaining any specific result *c* is bounded as

1 $$  \frac{p(\mathcal{M}(\mathcal{D}) = c)}{p(\mathcal{M}(\mathcal{D}') = c)} \le \exp(\epsilon).  $$

Because of similarity between $\mathcal {D}$ and $\mathcal {D}'$ the probabilities need to be similar to satisfy the condition. Differential privacy is preserved in post-processing, which makes it flexible to use in complex algorithms. The *ε* is a privacy parameter interpretable as a privacy budget, with higher values corresponding to less privacy preservation. Differentially private learning algorithms are usually based on perturbing either the input [5, 17], output [5, 18] or the objective [19, 20].

Here we apply differential privacy to regression. The aim is to learn a model to predict the scalar target *y*_*i*_ from *d*-dimensional inputs *x*_*i*_ (Fig. 1) as *y*_*i*_=*f*(*x*_*i*_)+*η*_*i*_, where *f* is an unknown mapping and *η*_*i*_ represents noise and modelling error. We wish to design a suitable structure for *f* and a differentially private mechanism for efficiently learning an accurate private *f* from a data set $\mathcal {D} = \{(x_{i}, y_{i})\}_{i=1}^{n}$.

We argue that a practical differentially private algorithm needs to combine two things: (i) it needs to provide *asymptotically efficiently private estimators* so that the excess loss incurred from preserving privacy will diminish as the number of samples *n* in the data set increases; (ii) it needs to *perform well on moderately-sized data*.

It was recently shown that perturbation of sufficient statisics of an exponential family model leads to asymptotically efficient differentially private Bayesian inference [21, 22]; to cover the second equally important criterion the methods of this paper are additionally needed. Asymptotic efficiency is nevertheless important because such methods always allow reaching stronger privacy with more samples.

While asymptotic efficiency is a nice guarantee, alone it is of little help for a specific learning problem with a fixed finite data set with size far from the asymptotic regime. It is difficult to prove the optimality of a method on finite data so performance needs to be studied empirically. We argue that for a method to perform well it needs to be designed to control the amount of shared private information. This has two components: (a) the dimensionality needs to be reduced, to avoid the inherent incompatibility of privacy and high dimensionality which has been discussed previously [23], and (b) robustness needs to be introduced by bounding and transforming each variable (feature) to a tighter interval. Controlling the amount of shared information also introduces a trade-off: compared to the non-private setting, decreasing the dimensionality may degrade the performance of the non-private approach (at least when reducing to a very low dimensionality), while a corresponding low-dimensional private algorithm may attain higher performance than a higher-dimensional one. This behaviour can be seen in the results of Fig. 6a where higher-dimensional differentially private algorithms perform worse than lower-dimensional ones, while for non-private algorithms a higher dimensionality would be better.

The essence of differential privacy is to inject a sufficient amount of noise to mask the differences between the computation results obtained from neighbouring data sets (differing by only one entry). The definition depends on the worst-case behaviour, which implies that suitably limiting the space of allowed results will reduce the amount of noise needed and potentially improve the results. In the output perturbation framework this can be achieved by bounding the possible outputs [18].

**Fig. 2:**
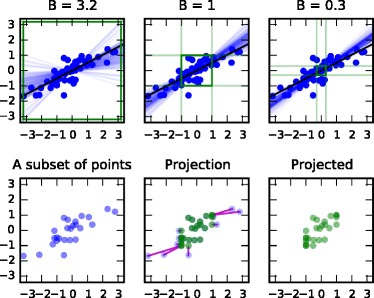
The effect of bounding data for differentially private learning of a regression model. Top: Bounding the data increasingly tightly (by B; green square) brings 1D robust private linear regression models (blue lines illustrating the distribution of results of the privacy-preserving algorithm) closer to the non-private model (black line) as less noise needs to be injected. Blue points: data. Bottom: The data are bounded in robust private linear regression by projecting outliers within the bounds (red lines; shown only for a subset of the points)

Here we propose a more powerful approach of bounding the data by projecting outliers to tighter bounds. The current standard practice in private learning is to linearly transform the data to desired bounds [20]. This is clearly sub-optimal as a few outliers can force a very small scale for the other points. Significantly higher signal-to-privacy-noise ratio can be achieved by setting the bounds to cover the essential variation in the data and projecting the outliers separately inside these bounds. This approach also robustifies the analysis against outliers as the projection can be made independent of the outlier scale. When applied to linear regression, we call the resulting model *robust private linear regression*. It is illustrated in Fig. 2.

### Algorithm overview

We incorporate differentially private learning into Bayesian linear regression. The linear regression model for scalar target *y*_*i*_, with *d*-dimensional input *x*_*i*_ and fixed noise precision *λ*, is defined by

2 $$\begin{array}{*{20}l} y_{i} | x_{i} &\sim N\left(x_{i}^{T} \beta, \lambda\right) \end{array} $$

3 $$\begin{array}{*{20}l} \beta &\sim N(0, \lambda_{0} I), \end{array} $$

where *β* is the unknown parameter to be learnt. The *λ* and *λ*_0_ are the precision parameters of the corresponding Gaussian distributions, and act as regularisers. Assuming the precision parameters are known and fixed, then given an observed data set $\mathcal {D} = \{(x_{i}, y_{i})\}_{i=1}^{n}$, all information about the data can be summarised by the sufficient statistics $n\overline {xx} = \sum _{i=1}^{n} x_{i} x_{i}^{T}$ and $n\overline {xy} = \sum _{i=1}^{n} x_{i} y_{i}$, which together with the prior completely determine the resulting posterior distribution. Instead of fixing the precision parameters, they can be assigned prior distributions. In that case, given an observed data set and sufficient statistics $n\overline {xx}$, $n\overline {xy}$ and $n\overline {yy}=\sum _{i=1}^{n} y_{i}^{2}$, we can use automatic differentiation variational inference (ADVI) [24] to fit a variational distribution to the posterior and then draw samples from the fitted distribution. We use ADVI because it gives similar results as Hamiltonian Monte Carlo sampling but significantly faster.

The robust private linear regression is based on perturbing these sufficient statistics. We use independent *ε*_*i*_-differentially private Laplace mechanisms [5] for perturbing each statistic with *ε*_*i*_=*p*_*i*_*ε* for each *i*=1,2,3 and *p*_1_+*p*_2_+*p*_3_=1. By the differential privacy composition theorem they together provide an *ε*-differentially private mechanism.

To improve the robustness of the method, we project the outliers in the private data sets to fit the data in the intervals [−*B*_*x*_,*B*_*x*_] and [−*B*_*y*_,*B*_*y*_]. A more detailed description of the learning is in “Methods” section.

## Results

### Optimal privacy budget split on synthetic data

**Fig. 3:**
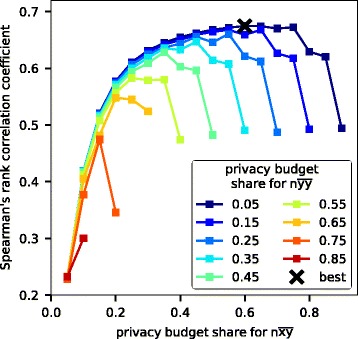
Optimal privacy budget split between sufficient statistics. Accuracy on a synthetic data set improves as a bigger proportion of the fixed privacy budget is assigned for $n\protect \overline {xy}$. The best performance is achieved by assigning term $n\protect \overline {yy}$ the smallest proportion 5%, term $n\protect \overline {xy}$ a large 60% proportion, and term $n\protect \overline {xx}$ the remaining 35% proportion of the privacy budget. Accuracy has been evaluated with 10-dimensional synthetic data, measured by Spearman’s rank correlation between the predicted and true values (higher values are better)

We find the optimal privacy budget split by generating an auxiliary data set of size *n* samples (here *n*=500) using the method described in “Methods” section. As illustrated in Fig. 3, the optimal split gives the largest proportion of the privacy budget to the term $n\overline {xy}$ (60%), the second largest proportion to the term $n\overline {xx}$ (35%), and the smallest possible proportion to the term $n\overline {yy}$ (5%).

### Effectiveness of data bounding on synthetic data

**Fig. 4:**
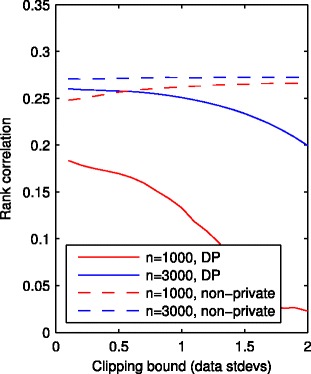
The effect of data bounding on regression model accuracy. The figure illustrates the effect of projecting the outliers to within the bounds in linear regression, for different sample sizes *n* with 10-dimensional synthetic data, evaluated by Spearman’s rank correlation between the predicted and true values (higher values are better), both for DP (solid lines) and non-private regression (dashed lines). The lines show a minor decrease in accuracy of the non-private algorithm as the projection threshold becomes increasingly tight. This minor decrease is eclipsed by a dramatic increase in the accuracy of the DP algorithm. Similar plots with higher dimensional data, and samples from a heavy-tailed distribution are included as Additional file 1: Figures S1 and S2

The importance of the projection is illustrated by the simulation results shown in Fig. 4. The simulation shows that aggressive projection can lead to clear improvement in the prediction accuracy. The figure shows the accuracy of simulated experiments as a function of the projection threshold represented as standard deviations away from the mean. As shown in the figure, the optimal threshold can be less than 0.5 sd away from the mean which implies that a significant majority of the data points get projected.

### Drug sensitivity prediction

**Methods** We applied the robust private linear regression model to predict drug sensitivity given gene expression data, in a setup where a small internal data set can be complemented by a larger set only available under privacy protection (Fig. 1). We used an experimental setting similar as in the recent DREAM-NCI drug sensitivity prediction challenge [25]; we also evaluate the results with the same measures, that is, Spearman’s rank correlation and weighted probabilistic concordance (wpc) index.

The data are from the Genomics of Drug Sensitivity in Cancer (GDSC) project [26, 27] (release 6.1, March 2017, http://www.cancerrxgene.org). Sensitivity measurements of 265 drugs for a panel of 985 human cancer cell lines are combined with gene expression data for the cell lines. The dimensionality of the RMA-normalised gene expression data was reduced from *d*=17,490 down to 64 based on prior knowledge about genes that are frequently mutated in cancer, provided by the GDSC project at http://www.cancerrxgene.org/translation/Feature. We further ordered the genes based on their mutation counts as reported at http://cancer.sanger.ac.uk/cosmic/curation. Drug responses were quantified by log-transformed IC50 values (the drug concentration yielding 50% response) from the dose response data measured at 9 different concentrations. The mean was first removed from each gene, *x*_*ij*_:=*x*_*ij*_−mean(*x*_1:*n*,*j*_), and each data point was then normalised to have L2-norm ∥*x*_*i*_∥_2_=1, which focuses the analysis on relative expression of the selected genes, and equalises the contribution of each data point. The mean was removed from drug sensitivities, *y*_*i*_:=*y*_*i*_−mean(*y*_1:*n*_).

The sensitivity to each drug was predicted with Bayesian linear regression. We compared the proposed robust private linear regression to state-of-the-art differentially private linear regression approaches that are based on output perturbation [18] and the functional mechanism [20]. Output-perturbed LR learns parameters *β* using the same LR model in Eq. (2), but instead of statistics the parameters are perturbed, in a data-independent manner. Our implementation of output-perturbed LR makes use of the minConf optimisation package [28]. For functional mechanism LR we used the code publicly available at https://sourceforge.net/projects/functionalmecha/.

We carried out a 50-fold Monte Carlo cross-validation process for different splits of the data set into train and test sets using different random seeds. For each repeat, we randomly split the 985 cell lines to two sets: 100 for testing and the rest for the training. We further randomly partitioned the training set to 30 non-private cell lines and used the rest as the private data set. In the experiments, we tested non-private data sizes from 0 to 30, and private data sizes from 100 to 800. After defining each split, the samples with missing drug responses were dropped, making the number of cell lines different across different drugs. The hyperparameters for the Gamma priors of precision parameters *λ*,*λ*_0_ in Eq. (9) were set to *a*=*b*=*a*_0_=*b*_0_=2. The Gamma(2,2) distribution has mean 1 and variance 1/2 and defines a realistic distribution over sensible values of precision parameters which should be larger than zero. We implemented the model and carried out the inference with the PyMC3 Python module [29]. Using ADVI, we fitted a normal distribution with uncorrelated variables to the posterior distribution. We computed the drug response predictions using *m*=5000 samples from the fitted variational distribution. The optimal privacy budget split was based on prediction performance averaged over five auxiliary data sets of 500 synthetic samples (approximately half of the GDSC data set size) and five generated noise samples, and for each split, the optimal projection thresholds were chosen similarly based on average performance over five auxiliary data sets and five noise samples. The prediction for each split was computed using *m*=5000 samples drawn from the variational distribution fitted with ADVI. The final optimal projection thresholds for each test case were chosen using the optimal budget split and based on average prediction performance over 20 auxiliary data sets and 20 noise samples. All auxiliary data sets were generated by fixing the precision parameter values to the prior means, *λ*=*λ*_0_=1. The prediction for each pair of projection thresholds was also computed using fixed precision parameters as in Eqs. (6) and (7), as generating samples from the fitted variational distribution for all test cases would have been infeasible in practice.

**Fig. 5:**
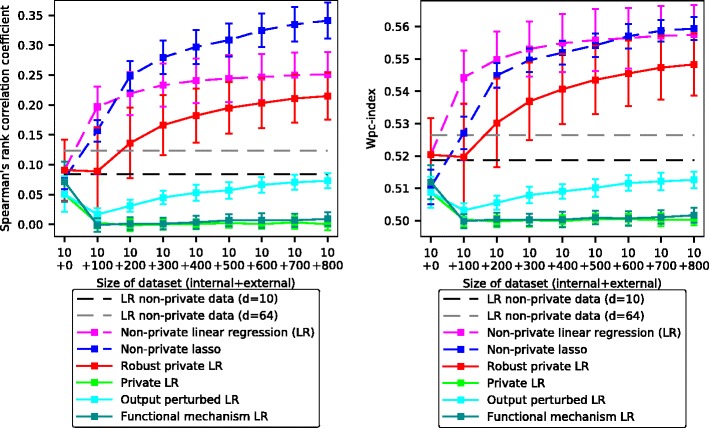
Accuracy of drug sensitivity prediction increases with amount of private data for the proposed robust private linear regression. The state-of-the-art methods fail to improve over just using the non-private data under strict privacy conditions, with reasonable data amounts. The baselines (horizontal dashed lines) are learned on 10 non-private data points; the private algorithms additionally have privacy-protected data (*x*-axis). The non-private algorithm (LR) has the same amount of additional non-privacy-protected data. Accuracy is measured by Spearman’s rank correlation coefficient over ranking cell lines by sensitivity to a drug (left; higher is better) and by weighted probabilistic concordance index (wpc-index; right; higher is better). All methods use 10-dimensional data except the gray baseline showing the best performance with 10 non-private 64-dimensional data points. Private methods use *ε*=2. Corresponding results for *ε*=1 are in Additional file 1: Figure S3 and results including non-private robust LR in Additional file 1: Figure S4. The results are averaged over all drugs and 50-fold Monte Carlo cross-validation; error bars denote standard deviation over 50 Monte Carlo repeats

**Results** The prediction accuracies of the compared methods are illustrated in Fig. 5. Unlike the earlier differentially private methods, the proposed robust private linear regression can improve the prediction accuracy (ranking of new cell lines [25] to sensitive vs insensitive as measured by Spearman’s rank correlation and the wpc-index) over the baseline of using only a small internal data set, when feasible amounts of privacy protected data are received. The output-perturbed linear regression is able to learn something from the private data too, but its performance is significantly worse than with the proposed approach. Results with more stringent privacy (*ε*=1 instead of *ε*=2) in Additional file 1: Figure S3 show overall lower accuracy for the private methods but are again qualitatively similar.

The comparison includes non-private lasso regression which was the best-performing method in the DREAM drug sensitivity prediction challenge [25] using only expression data. Non-private lasso regression is clearly superior to the other methods for Spearman’s rank correlation. With the more relevant wpc-index, non-private linear regression is on par with non-private lasso regression and the proposed robust private linear regression is quite close behind. Overall, our differentially private method using 800 samples is on par with non-private lasso regression with 200 samples, suggesting we can match the accuracy of the state-of-the-art non-private predictions under differential privacy with a reasonable increase in the number of samples needed. The good performance of the lasso regression which ultimately uses a linear model also suggests that with better feature selection, private linear regression could potentially do even better.

Among the state-of-the-art differentially private algorithms, the output perturbation method [18] is the most accurate one, but it is still significantly less accurate than the proposed method. The relatively poor performance of the output perturbation method on our benchmark compared to their previously reported results is due to the difficulty and higher dimensionality of our prediction task.

**Fig. 6:**
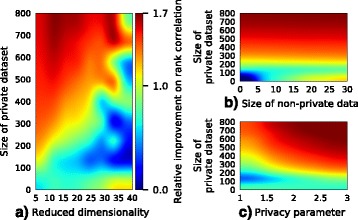
Key trade-offs in differentially private learning. Relative improvements over baseline (10 non-private data points). **a**, As the dimensionality increases, more data are needed to improve performance of the private methods. **b**, With enough private data, adding more non-private data does not significantly increase the performance. **c**, More data are needed if privacy guarantees are tighter (privacy parameter *ε* is smaller). Data dimensionality is 10, the size of non-private data is 10, and *ε*=2 (except when otherwise noted)

To improve prediction performance in differentially private learning, trade-offs need to be made between dimensionality and the amount of data (Fig. 6a), and between the strength of privacy guarantees and the amount of data (Fig. 6c). In our experiments the amount of optional non-private data matters significantly only when there is very little private data (Fig. 6b), which is probably due to the fact that every sample gets equal weight in the model regardless of its origin.

To understand the reason for the success of the robust private linear regression, we also tested it without the projection step. The proposed non-linear projection of the data to tighter bounds is clearly the key to the success of the method, as without it the method performs very poorly (green line for Private LR in Fig. 5), and is not able to improve prediction performance using the available data. Because of the different formulations they are based on, the alternative differentially private algorithms considered here cannot directly benefit from the projection to decrease the amount of injected noise and hence would not experience a similar improvement in accuracy.

The effect of the projection is studied further in Additional file 1: Figure S4 which includes a non-private robust linear regression using the projection approach. The performance of this approach is slightly worse than that of the regular linear regression. This verifies our assumption that best private learning methods are not direct translations of best non-private methods but new methods that take into account the privacy constraints.

## Discussion

A key question which needs to be answered before applying differentially private methods in practical personalised medicine, is whether they can compromise patient safety as previously warned [14]. If there are sufficient amounts of data available without restrictions on their use, the more accurate non-private methods are certainly preferable. However, we believe in more realistic scenarios the amount of non-restricted data is limited, and larger sets are only available under privacy restrictions. As demonstrated by our results, the proposed differentially private methods can provide more accurate predictions in this case. Furthermore, because of the asymptotic efficiency of the method, the extra “price” for privacy diminishes as the size of the data set increases.

At the heart of any privacy-aware data analysis is a trade-off between privacy and utility of the data in the analysis. The only way to ensure perfect privacy is not to use the data at all, which corresponds to zero utility. The interesting question is how much utility can be obtained under reasonable privacy guarantees. Asymptotically efficient differentially private methods always allow reaching a utility arbitrarily close to that of the corresponding non-private model by adding more samples. In the context of the results reported here the asymptotic efficiency of the method means that larger data sets available in the future will allow higher prediction accuracy, stronger privacy guarantees or some combination of both.

The modelling setup of Fig. 1 and the ability of our method to effectively combine data sets under different privacy requirements creates a promise of new methods of sharing and utilising privacy-sensitive data. Because there have not been learning algorithms capable of leveraging on privacy-protected data sets, data owners have not had reasons to share data in a privacy-protected manner. Now we hope that new methods, including the ones presented in this paper, will help motivate more differentially private data sharing that can then in turn increase their utilisation, which will enable better predictions and further better healthcare and services even more generally.

In this paper we have focused on scalar targets in regression. There is a trivial extension of the same algorithm that yields a (*q*+1)*ε*/2-differentially private algorithm for *q*-dimensional targets, which is non-ideal when *q* is large. A simple way around this is to increase the number of samples as doubling the number of samples allows halving *ε* with equivalent accuracy. Still, careful selection of which targets to model or some dimensionality reduction in the target space will likely be useful for large *q*.

Robust private linear regression treats non-private and scrambled private data similarly in the model learning. An interesting next step for further improving the accuracy on very small private data would be to give different weights to the clean and privacy-scrambled data by incorporating knowledge of the injected noise in the Bayesian inference, as has been proposed for generative models [30], but which is non-trivial in regression.

## Conclusions

We presented methodology that can make use of personal genomic data for precision medicine modelling under a strict differential privacy quarantee. Through improvements in the previously unappreciated data scaling and projection, the simple-to-implement method constitutes a foundation for designing practical differentially private learning methods. We were able to obtain dramatically more accurate predictions in the very challenging drug sensitivity prediction task, utilising moderate-sized, privacy-protected data. Moreover, being asymptotically efficient, the loss in performance relative to non-private approach will diminish as the amount of data grows. The differentially private modelling will likely have a significant impact not only in precision medicine but also in machine learning more generally and change the way sensitive data are stored and utilised.

## Methods

### Robust private linear regression

We project the outliers in the private data sets to fit the data in the intervals [−*B*_*x*_,*B*_*x*_] and [−*B*_*y*_,*B*_*y*_] as

4 $$\begin{array}{*{20}l}  x_{ij} &= \max(-B_{x}, \min(x_{ij},B_{x}))  \\ y_{i} &= \max(-B_{y}, \min(y_{i},B_{y})). \end{array} $$

After the projection, ∥*x*_*i*_∥_*∞*_≤*B*_*x*_ and |*y*_*i*_|≤*B*_*y*_, where ∥*x*_*i*_∥_*∞*_= max*jx*_*ij*_. We add noise to the sufficient statistics as

5 $$\begin{array}{*{20}l}  n\overline{xx} + L_{1},\: L_{1} &\sim \text{Laplace}\left(0,\frac{(d^{2}+d)B_{x}^{2}}{p_{1}\epsilon}\right)  \\ n\overline{xy} + L_{2},\: L_{2} &\sim \text{Laplace}\left(0,\frac{2{dB}_{x}B_{y}}{p_{2}\epsilon}\right)  \\ n\overline{yy} + L_{3},\: L_{3} &\sim \text{Laplace}\left(0,\frac{B_{y}^{2}}{p_{3}\epsilon}\right). \end{array} $$

This generalises earlier work on bounded variables [21] to the unbounded case by introducing the projection. It can be shown that this yields a valid differentially private mechanism (Additional file 1: Supplementary Information).

### Posterior inference and prediction

If the precision parameters *λ* and *λ*_0_ are assumed to be known and fixed, then given an observed data set $\mathcal {D} = \{(x_{i}, y_{i})\}_{i=1}^{n}$ with sufficient statistics $n\overline {xx} = \sum _{i=1}^{n} x_{i} x_{i}^{T}$ and $n\overline {xy} = \sum _{i=1}^{n} x_{i} y_{i}$, the posterior distribution of *β* is Gaussian, $p(\beta | \mathcal {D}) = N(\beta ;\; \mu _{*}, \Lambda _{*})$, with precision

6 $$ \Lambda_{*} = \lambda_{0} I + \lambda n\overline{xx}  $$

and mean

7 $$ \mu_{*} = \Lambda_{*}^{-1} (\lambda n\overline{xy}).  $$

After learning with the training data set $\mathcal {D}_{\text {train}}$, the prediction of *y*_*i*_ given *x*_*i*_ is computed as follows:

8 $$ \hat{y}_{i} = x_{i}^{T} \mu_{*}.  $$

A more robust alternative is to define prior distributions for the precision parameters. In our case, a Gamma prior is assigned for both:

9 $$\begin{array}{*{20}l} \lambda &\sim \text{Gamma}(a,b)\\ \lambda_{0} &\sim \text{Gamma}(a_{0},b_{0}). \end{array} $$

A variational normal distribution is fitted to the posterior with ADVI. The precision parameters and correlation coefficients *β* are then sampled from the fitted distribution. For this purpose, the data likelihood in Eq. (2) needs to be expressed in terms of the sufficient statistics $n\overline {xx}$, $n\overline {xy}$ and $n\overline {yy}=\sum _{i=1}^{n} y_{i}^{2}$, which results in

10 $$ p(y|X,\beta,\lambda)\,=\, \left(\!\frac{\lambda}{2\pi}\!\right)^{n/2} \!\exp\left(\!-\frac{\lambda}{2}\left(\beta^{T} n\overline{xx}\beta \,-\, 2\beta^{T} n\overline{xy} + n\overline{yy}\right)\right).\\  $$

The prediction of *y*_*i*_ is computed using *x*_*i*_ and averaging over a sufficiently large number *m* of sampled regression coefficients *β*^(*k*)^ as

11 $$ \hat{y}_{i} = \int p(y|\beta,X_{\text{test},i})p(\beta|\mathcal{D}_{\text{train}})d\beta \approx \frac{1}{m} \sum_{k=1}^{m} x_{\text{test},i}^{T} \beta^{(k)}.  $$

For evaluation we keep a part of the data set $\mathcal {D}$ aside as $\mathcal {D}_{\text {test}}$ (not used for training), and after predicting $\hat {y}_{i}$, we evaluate the error between the actual *y*_test,*i*_ and $\hat {y}_{i}$. In this paper, we do this using Spearman’s rank correlation coefficient to evaluate how well the predictions separate sensitive and insensitive cell lines.

### Determining the privacy budget split and projection thresholds

The privacy budget proportions *p*_1_, *p*_2_, *p*_3_ and the projection thresholds *B*_*x*_ and *B*_*y*_ are important parameters for good performance. We propose finding the optimal parameter values on an auxiliary synthetic data set of the same size, which was found to be effective in our case. We generate the auxiliary data set of *n* samples using a generative model similar to the one specified in Eq. (2):

12 $$\begin{array}{*{20}l} x_{i} & \sim N(0, I)  \\ y_{i} | x_{i} &\sim N\left(x_{i}^{T} \beta, \lambda\right)  \\ \beta & \sim N(0, \lambda_{0} I), \end{array} $$

where *d* is the dimension.

For all possible combinations of (*p*_1_,*p*_2_,*p*_3_)∈{0.05,0.1,…,0.90}^3^, where *p*_1_+*p*_2_+*p*_3_=1, we project the data using thresholds for the current split, and we perturb the sufficient statistics according to the current budget split. We compute the prediction as in Eq. (11) using samples drawn from the variational distribution fitted with ADVI and compute the error with respect to the original values. The error measure we use is Spearman’s rank correlation between the original and predicted values. The split which gives the minimum error is used in all test settings.

We parametrise the projection thresholds as a function of the data standard deviation as

13 $$\begin{array}{*{20}l} & B_{x}= \omega_{x} \sigma_{x},\quad B_{y}= \omega_{y} \sigma_{y} \end{array} $$

14 $$\begin{array}{*{20}l} & \omega_{x},\omega_{y} \in \{0.1\omega\}_{\omega=1}^{20}, \end{array} $$

where the *σ*_*x*_ and *σ*_*y*_ are the standard deviations of *x* (considering all dimensions) and *y*, respectively. With all 400 pairs of (*B*_*x*_,*B*_*y*_) as specified above, we apply the outlier projection method of Eq. (4). We fit the model using the projected values and then compute the error with respect to the original values. The pair of (*ω*_*x*_,*ω*_*y*_) which gives the minimum error is used to define the (*B*_*x*_,*B*_*y*_) for the real data as in Eq. (13). As the error we used Spearman’s rank correlation between original *y*_1:*n*_ and predicted $\hat {y}_{1:n}$ based on the model learnt with projected values.

### Combining internal and external data sets

Our modelling setup (Fig. 1) allows combining non-private data (also called internal data) with data requiring privacy protection. Multiple data sets can be combined in the Bayesian modelling framework by adding the sufficient statistics $n\overline {xx}$, $n\overline {xy}$ and $n\overline {yy}$ arising from various data sets together to produce aggregate sufficient statistics for the combined data. Data sets requiring privacy protection can be protected by adding noise to the corresponding sufficient statistics as described.

### Algorithm details

We first determine the optimal budget split *p*_1_, *p*_2_, *p*_3_ and then choose the optimal parameters *ω*_*x*_, *ω*_*y*_ using the synthetic auxiliary data method as described above. We test the algorithm using Monte Carlo cross-validation. For each repeat, we normalise the data and compute the standard deviation *σ*_*x*_ of the input data and *σ*_*y*_ of the target data from the normalised private data set. The projection thresholds *B*_*x*_, *B*_*y*_ are then computed as in Eq. (13) and both the private and non-private training data are projected using the same acquired thresholds as in Eq. (4). The prediction for the test data is computed from ADVI samples as in Eq. (11). The precision is computed between the predicted and actual *y*_*i*_ for the test data.

### Alternative interpretation: transformed linear regression

The outlier projection mechanism can also be interpreted to produce a transformed linear regression problem,

15 $$ \phi_{y}(y_{i}) | x_{i} \sim N\left(\phi_{x}(x_{i})^{T} \beta, \lambda\right),  $$

where the functions *ϕ*_*y*_() and *ϕ*_*x*_() implementing the outlier projection can be defined as

16 $$\begin{array}{*{20}l} \phi_{y}(y_{i}) &= \max(-B_{y}, \min(B_{y}, y_{i})) \end{array} $$

17 $$\begin{array}{*{20}l} \phi_{x}(x_{i}) &= \max(-B_{x}, \min(B_{x}, x_{i})). \end{array} $$

The normalisation of data can also be included as a transformation. This interpretation makes explicit the flexibility in designing the transformations: the differential privacy guarantees will remain valid as long as the transformations obey the bounds

18 $$  \phi_{y}(y_{i}) \in \left[-B_{y}, B_{y}\right], \quad \phi_{x}(x_{i}) \in \left[-B_{x}, B_{x}\right].  $$

## Reviewers’ comments

### Reviewer’s report 1: Zoltan Gaspari, Pazmany Peter Catholic University, Hungary

While the manuscript might be of interest to the statistics community, in its present form it seems to provide little biological significance. The paper describes how the sensitivity of different linear regression models changes as a function of the amount of anonymized data. The fact that drug-sensitivity data are used is merely a technical choice, the manuscript provides no novel insights and the obtained rank correlations (on real data) seem to be irrelevant even in the best cases.

Authors’ response:*We wish to thank the reviewer for expressing his opinion but respectfully disagree. Far from being merely a technical choice, solving the drug sensitivity prediction task was our primary motivation when developing the method, and we strongly believe the drug sensitivity modelling community would benefit significantly from the work as a proof of principle that this kind of privacy-preserving modelling is possible. This finding could have far-reaching implications to future data generation and sharing for similar tasks, given the privacy concerns with broad availablity of human genomic data. Given the risk of model inversion attacks, even highly refined published models carry a risk of leaking private data used in the training.*

It is not clear how the method relates to previously published ones (http://www.nature.com/nbt/journal/v32/n12/fig_tab/nbt.2877_T1.html?foxtrotcallback=true) and whether it is comparable to those at all.

Authors’ response: *The results are not directly comparable because we are using a different data set with more samples but fewer features. We have now added a new comparison to non-private lasso regression that was the best method using only expression data in the DREAM challenge (linked above). As shown in Fig.* 5, *non-private lasso regression performs really well on the Spearman’s rank correlation, but with the more relevant weighted probabilistic concordance index its results are quite similar to non-private linear regression and our private method is not too far behind. In summary, our differentially private method using 800 samples is on par with non-private lasso regression with 200 samples, suggesting we can match the accuracy of the state-of-the-art non-private predictions under differential privacy with a reasonable increase in the number of samples needed.*

The biological relevance of the bounding of the values and the omission of data in order to reduce the dimensions should also be justified. It is not at all trivial that these steps are allowed without losing relevant biological information and insights.

Authors’ response: *All models are simplifications of the world and ours is no different. Interpretability and prediction accuracy of a model are often at odds. We believe our model attains a good compromise in this respect because ultimately we only combine non-linear clipping transformations of scalar variables with easily interpretable linear regression. Finding new and even better compromises that yield accurate predictions while maintaining even higher biological interpretability is an interesting avenue for future research.*

I recommend that the work should be presented in a way that allows the judgment of the biological relevance of the resulting analysis and the possible loss of information introduced by the transformations. It is highly desirable that the description of the approach includes a case with real data where both retaining the biological significance and the privacy issues can be clearly and effectively shown.

Authors’ response: *As noted above, we have added a new comparison with the top-performing method from the DREAM challenge using only expression data. All experiments have been performed with the largest available collection of real data, so we believe we are addressing the question as well as possible without extensive and very expensive new data collection.*

### Reviewer’s report 2: David Kreil, University of Warwick, UK

Increasingly, there are not just academic analyses but also public concerns about the privacy of patient data. For instance, data sharing arrangements between a company developing modern algorithms for precision medicine (DeepMind) and a group of hospitals of the U.K. National Health Service were vocally objected, with the privacy of patient data questioned in the public press [*]. Especially in this context, the recent work of Honkela et al. reported in their manuscript on Efficient differentially private learning improves drug sensitivity prediction are of general interest and may have substantial impact beyond their immediate field of research.

Machine learning algorithms preserving differential privacy need to strike many trade-offs, and the development of approaches that guarantee some degrees of privacy while inferring accurate models for prediction is a novel and highly active field of research [**]. Established approaches include randomly perturbing the input, the objective, or the output of a model in training. Besides questions of privacy guarantees and learning efficiency, there is a practical aim of effectively exploiting a combination of private and public data sets with the hope of deriving better models than can be learned from public data alone. The authors seek to address this challenge in the context of linear regression models.

It would be interesting if the authors could relate their analysis to prior work looking into combining public and private data, such as distributed differentially private regression [***].

Authors’ response: *Thanks for pointing this out. Ji et al. [15] have combined public and private data in a different problem, using a naive algorithm. They have a clever idea of only using the public data to compute the Hessian matrix needed for Newton–Raphson optimisation of logistic regression as this can be more sensitive to the DP noise, but otherwise the algorithm is highly suboptimal and the classification accuracy is not high. We have now discussed this at the end of*Background *section.*

The authors propose and assess a novel feature mapping that clips extreme data values to specified bounds. Together with adding noise to a set of sufficient statistics, this yields a differentially private mechanisms (as shown in a Supplement to an identically entitled arxiv deposition of the authors). The analysis then proceeds to characterize this approach, both in terms of the response to parameter choices for the method as well as its properties for different private and public data set sizes. Nicely, this clipping of unusual data points reduces the amount noise that the differentially private regression mechanism requires to meet its privacy guarantees. The authors emphasize that while the method performs better with more data as required, they already obtain good results for realistic, reasonably small data sizes. For sufficiently large private data sets, the relative penalty for differential privacy begins to vanish. The authors take great care in determining method parameters in a principled way, examining robustness, and cross-validating their results. While the simulations to determine an optimal privacy budget splice between the different sufficient statistics may use data that look different to ‘real’ data, this will not affect the validity of the subsequent characterization of their method. If anything, conclusions will be conservative. The real-world data used for characterizing their approach make use of a recent release of the Genomics of Drug Sensitivity in Cancer (GDSC) project, and thus an up-to-date and topical use case is employed.

It might in addition also be interesting to see how strongly performance varies for different kinds of data and regression problems to examine the effects of domain specific types of noise (more or less heavy tailed), biases and correlations in the data, as well as the effects of the dimensionality of the regressors.

Authors’ response: *We have studied the performance of the proposed method using synthetic data, both higher-dimensional data and also using a Student’s t distribution with 1 degree of freedom, which has much heavier tails than the normal distribution. We have included two figures in the Supplementary corresponding to these two experiments. The outcome of these experiments regarding the effect of bounding threshold on data samples is similar to results in the main text (Fig.* 4), *but with different curvatures.*

The authors in conclusion raise the possibility of future follow-up work on further improving the algorithm’s promising performance on very small private data sets. The authors largely evaluate performance for a privacy parameter epsilon =2. Their Fig. 6c explores a range of epsilon =1..3. While other methods have already been failing for higher epsilon =5, Wu et al. [17] have shown promising regression results for epsilon as low as 0.1. What do the authors observe for their approach for such low privacy budgets and reasonable data set sizes (assuming patient numbers are fixed within a range as shown in Fig. 5)?

Authors’ response: *We have compared with the method by [17] (now [18]) in our experiments: output perturbed LR (the blue curve in Fig.* 5). *Among the state-of-the-art differentially private algorithms, the output perturbation technique by [18] is the most accurate one, but it is still clearly inferior compared to the proposed method. It is worth noting that [18] was able to achieve a very low mean squared error (MSE) over parameters on a very different dataset. Our dataset is quite different and more challenging, for example due to higher dimensionality. Our evaluation metric corresponds to the one used in the DREAM challenge and is more relevant to the task than MSE. We have included this discussion in the*“Results” *section.*

Further to Fig. 5, I was struck by the relatively low correlation coefficients achieved (0.1..0.3) even without guarantees of differential privacy. This contrasts with the much higher values achieved in simulation (0.7, Fig. 3). If that is to be expected for these data, is Spearman rank correlation perhaps not an ideal measure for prediction performance?

Authors’ response: *We have considered the evaluation policy by [25] to use wpc-index and Spearman’s rank correlation coefficient. We believe these metrics focus better on the task of distinguising between suitable and unsuitable drugs for a particular patient instead of wasting modelling effort on predicting specific effective concentrations. The updated manuscript uses both metrics more evenly.*

Finally, what are the authors’ thoughts regarding the challenge of model inversion attacks with improved model quality, as also discussed by Wu et al. [17]? Do the robustification / bounding steps potentially contribute to alleviating this issue somewhat?

Authors’ response: *From the study by Wu et al. [17] (now [18]), it is evident that vulnerability to inversion attacks is correlated with the privacy budget. That is, with lower value of the differential privacy parameter* (*ε*) *the model is more robust to attacks. This is a major motivation for our work; to find maximally accurate models that work with as small* *εas possible. Detailed study of model inversion attacks for drug sensitivity prediction is an important topic for future work.*

References [*] https://www.cnbc.com/2017/07/03/google-deepmind-nhs-deal-health-data-illegal-ico-says.html [**] Aldeen et al. A comprehensive review on privacy preserving data mining. SpringerPlus. 2015, and Dwork & Roth. The Algorithmic Foundations of Differential Privacy. FnT-TCS. 2014 [***] Ji Z, Jiang X, Wang S, Xiong L, and L Ohno-Machado. (2014) Differentially private distributed logistic regression using private and public data. BMC Medical Genomics 7, S14. Numbered citations are to references cited in the original manuscripts itself.

Editorial and minor points: The manuscript should be self-sufficient, so instead of citing the Supplement of their identically titled arxiv deposition that provides further methodological, I think it would be better if the authors could please add this information to the Additional file 1 or appendix of this manuscript.

Authors’ response: *We have included the information as Additional file* 1*to the current paper.*

Page 2 “symmetry between D and” → “similarity”? Page 3 “we can automatic differentiation” → “we can use...”? Please introduce variables and symbols on first use; it may also be helpful for some readers to define the norm “ ∥*x*_*i*_∥_*∞*_= max*i*(|*x*_*i*_|)” on page 7 Figures should be shown and numbered in the order in which they are referenced in the text. Currently, the second figure referenced is Fig. 6.

Authors’ response: *We have rectified all of the above issues.*

## Additional file

Additional file 1 Supplementary Information for “Efficient differentially private learning improves drug sensitivity prediction”. (PDF 342 kb)

## Abbreviations

ADVI: Automatic differentiation variational inference
DP: Differential privacy
GDSC: Genomics of drug sensitivity in cancer
LR: Linear regression
