# Supplementary material for: Efficient differentially private learning improves drug sensitivity prediction
Source: Biol Direct. 2018 Feb 6;13:1. doi: 10.1186/s13062-017-0203-4 (PMC5801888; doi:10.1186/s13062-017-0203-4)
Supplement: Additional file 1 — Supplementary Information for “Efficient differentially private learning improves drug sensitivity prediction”. (PDF 342 kb) [file 13062_2017_203_MOESM1_ESM.pdf]

Supplementary Information for "Efficient differentially private learning improves drug sensitivity prediction"

Antti Honkela, Mrinal Das, Arttu Nieminen, Onur Dikmen & Samuel Kaski

1 Theoretical background

We argue that effective differentially private predictive modelling methods can be developed by a combination of:

i. An asymptotically efficiently private mechanism for which the effect of the noise added to guarantee privacy vanishes as the number of samples increases; and

ii. A way to limit the amount of private information to be shared. This yields better performance on finite data as less noise needs to be added for equivalent privacy. This can be achieved through a combination of two things:

   a. An approach to decrease the dimensionality of the data prior to the application of the private algorithm; and

   b. A method to focus the privacy guarantees to relevant variation in data.

Criterion i can be formally stated through additional loss in accuracy or utility of the estimates because of privacy. Our main asymptotic result is that the optimal convergence rate of a differentially private mechanism to a Bayesian estimate is $\mathcal{O}(1/n)$, which can be reached by our proposed mechanism.

Criterion ii is non-asymptotic and thus more difficult to address theoretically. It manifests itself in the constants in the convergence rates as well as empirical findings on the effect of dimensionality reduction and projecting outliers to tighter bounds as discussed in the main text and in Fig. 2.

1.1 Definition of asymptotic efficiency

We begin by formalisation of the theory behind Criterion i.

Definition 1. A differentially private mechanism $\mathcal{M}$ is *asymptotically consistent with respect to an estimated parameter* θ if the private estimates $\hat{\theta}_{\mathcal{M}}$ given a data set $\mathcal{D}$ converge in probability to the corresponding non-private estimates $\hat{\theta}_{NP}$ as the number of samples, $n = |\mathcal{D}|$, grows without bound, i.e., if for any[1] $\alpha > 0$,

$$\lim_{n\to\infty} \Pr\{\|\hat{\theta}_{\mathcal{M}} - \hat{\theta}_{NP}\| > \alpha\} = 0.$$

Definition 2. A differentially private mechanism $\mathcal{M}$ is *asymptotically efficiently private with respect to an estimated parameter* θ, if the mechanism is asymptotically consistent and the private estimates $\hat{\theta}_{\mathcal{M}}$ converge to the corresponding non-private estimates $\hat{\theta}_{NP}$ at the rate $\mathcal{O}(1/n)$, i.e., if for any $\alpha > 0$ there exist constants C, N such that

$$\Pr\{\|\hat{\theta}_{\mathcal{M}} - \hat{\theta}_{NP}\| > C/n\} < \alpha$$

for all $n \geq N$.

The term asymptotically efficiently private in the above definition is justified by the following theorem, which shows that the rate $\mathcal{O}(1/n)$ is optimal for estimating expectation parameters of exponential family distributions. As it seems unlikely that better rates could be obtained for more difficult problems, we conjecture that this rate cannot be beaten for Bayesian estimates in general.

Theorem 1. *The private estimates $\hat{\theta}_{\mathcal{M}}$ of an exponential family posterior expectation parameter θ, generated by a differentially private mechanism $\mathcal{M}$ that achieves ϵ-differential privacy for any $\epsilon > 0$, cannot converge to the corresponding non-private estimates $\hat{\theta}_{NP}$ at a rate faster than $1/n$. This is, assuming $\mathcal{M}$ is ϵ-differentially private, there exists no function $f(n)$ such that* $\limsup nf(n) = 0$ *and for all $\alpha > 0$, there exists a constant N such that*

$$\Pr\{\|\hat{\theta}_{\mathcal{M}} - \hat{\theta}_{NP}\| > f(n)\} < \alpha$$

for all $n \geq N$.

Proof. The non-private estimate of an expectation parameter of an exponential family is [1]

$$\hat{\theta}_{NP}|x_1, \dots, x_n = \frac{n_0 x_0 + \sum_{i=1}^{n} x_i}{n_0 + n}. \tag{1}$$

The difference of the estimates from two neighbouring data sets differing by one element is

$$(\hat{\theta}_{NP}|\mathcal{D}) - (\hat{\theta}_{NP}|\mathcal{D}') = \frac{x - y}{n_0 + n}, \tag{2}$$

[1]We use α in limit expressions instead of usual ϵ to avoid confusion with ϵ-differential privacy.

where x and y are the corresponding mismatched elements. Let $\Delta = \max(\|x - y\|)$, and let $\mathcal{D}$ and $\mathcal{D}'$ be neighbouring data sets including these maximally different elements.

Let us assume that there exists a function $f(n)$ such that $\limsup nf(n) = 0$ and for all $\alpha > 0$ there exists a constant N such that

$$\Pr\{\|\hat{\theta}_{\mathcal{M}} - \hat{\theta}_{NP}\| > f(n)\} < \alpha$$

for all $n \geq N$.

Fix $\alpha > 0$ and choose $M \geq \max(N, n_0)$ such that $f(n) \leq \Delta/4n$ for all $n \geq M$. This implies that

$$\|(\hat{\theta}_{NP}|\mathcal{D}) - (\hat{\theta}_{NP}|\mathcal{D}')\| = \frac{\Delta}{n_0 + n} \geq \frac{\Delta}{2n} \geq 2f(n). \tag{3}$$

Let us define the region $C_{\mathcal{D}} = \{t \mid \|(\hat{\theta}_{NP}|\mathcal{D}) - t\| < f(n)\}$. Based on our assumptions we have

$$\Pr(\hat{\theta}_{\mathcal{M}}|\mathcal{D} \in C_{\mathcal{D}}) > 1 - \alpha \tag{4}$$

$$\Pr(\hat{\theta}_{\mathcal{M}}|\mathcal{D}' \in C_{\mathcal{D}}) < \alpha \tag{5}$$

which implies that

$$\frac{\Pr(\hat{\theta}_{\mathcal{M}}|\mathcal{D} \in C_{\mathcal{D}})}{\Pr(\hat{\theta}_{\mathcal{M}}|\mathcal{D}' \in C_{\mathcal{D}})} > \frac{1 - \alpha}{\alpha} \tag{6}$$

which means that $\mathcal{M}$ cannot be differentially private with $\epsilon < \log((1 - \alpha)/\alpha) \to \infty$ as $\alpha \to 0$. □

1.2 Different utility functions

Definition 3. Let $\mathcal{U}(\hat{\theta}_{NP}(\mathcal{D}))$ measure the utility of the non-private model $\hat{\theta}_{NP}$ estimated from data set $\mathcal{D}$ and let $\mathcal{U}(\hat{\theta}_{\mathcal{M}}(\mathcal{D}))$ measure the corresponding utility of the private model $\hat{\theta}_{\mathcal{M}}$ obtained using differentially private mechanism $\mathcal{M}$. The mechanism $\mathcal{M}$ is *asymptotically consistent with respect to a bounded utility* $\mathcal{U}$ if the random variables $\mathcal{U}(\hat{\theta}_{\mathcal{M}}(\mathcal{D}))$ converge in probability to $\mathcal{U}(\hat{\theta}_{NP}(\mathcal{D}))$ as the number of samples, $n = |\mathcal{D}|$, grows without bound, i.e., if for any $\alpha > 0$,

$$\lim_{n\to\infty} \Pr\{|\mathcal{U}(\hat{\theta}_{\mathcal{M}}(\mathcal{D})) - \mathcal{U}(\hat{\theta}_{NP}(\mathcal{D}))| > \alpha\} = 0.$$

Theorem 2. *A differentially private mechanism $\mathcal{M}$ that is asymptotically consistent with respect to a set of parameters is asymptotically consistent with respect to any continuous utility that only depends on those parameters.*

Proof. If $\hat{\theta}_{\mathcal{M}}$ converges in probability to $\hat{\theta}_{NP}$ then by the continuous mapping theorem the value of $\mathcal{U}(\hat{\theta}_{\mathcal{M}})$ will converge in probability to $\mathcal{U}(\hat{\theta}_{NP})$. □

1.3 Example: Gaussian mean

Theorem 3. *Differentially private inference of the mean of a Gaussian variable, with Laplace mechanism to perturb the sufficient statistics, is asymptotically consistent with respect to the posterior mean.*

Proof. Let us consider the model

$$x_i \sim N(\mu, \Lambda)$$
$$\mu \sim N(\mu_0, \Lambda_0)$$

with μ as the unknown parameter and Λ and Λ_0 denoting the fixed prior precision matrices of the noise and the mean, respectively. We assume $||x_i||_1 \leq B$ and enforce this by projecting the larger elements to satisfy this bound.

Let the observed data set be $\mathcal{D} = \{x_i\}_{i=1}^n$ with sufficient statistic $n\bar{x} = \sum_{i=1}^n x_i$.

The non-private posterior mean is

$$\mu_{NP} = (\Lambda_0 + n\Lambda)^{-1}(\Lambda n\bar{x} + \Lambda_0\mu_0).$$

The corresponding private posterior mean is obtained by replacing $n\bar{x}$ with the perturbed version $n\bar{x}' = n\bar{x} + \delta$, where $\delta = (\delta_1, \dots, \delta_d)^T \in \mathbb{R}^d$ with $\delta_j \sim \text{Laplace}(0, \frac{2Bd}{\epsilon})$ and $d = \dim(x_i)$, yielding

$$\mu_{DP} = (\Lambda_0 + n\Lambda)^{-1}(\Lambda(n\bar{x} + \delta) + \Lambda_0\mu_0).$$

The difference of the private and non-private means is

$$\begin{aligned} \|\mu_{DP} - \mu_{NP}\|_1 &= \|(\Lambda_0 + n\Lambda)^{-1}(\Lambda\delta)\|_1 \\ &= \|(\Lambda^{-1}\Lambda_0 + n \cdot I)^{-1}\delta\|_1 \leq \frac{c}{n}\|\delta\|_1, \end{aligned}$$

which is valid for all $c > 1$ for large enough n. This implies that

$$\Pr\{\|\mu_{DP} - \mu_{NP}\|_1 \geq \alpha\} \leq \Pr\left\{\frac{c}{n}\|\delta\|_1 \geq \alpha\right\} \to 0$$

as $n \to \infty$ for all $\alpha > 0$. □

Theorem 4. *Differentially private inference of the mean of a Gaussian variable with Laplace mechanism to perturb the input data set (naive input perturbation) is* not *asymptotically consistent with respect to the posterior mean.*

Proof. The mechanism is almost the same as in Theorem 3, but we now have $n\bar{x}' = n\bar{x} + \sum_{i=1}^n \delta_i)$ where $\delta_i = (\delta_{i1}, \dots, \delta_{id})^T \in \mathbb{R}^d$ with $\delta_{ij} \sim$

Laplace$(0, \frac{2Bd}{\epsilon})$. Similar computation as above yields

$$\|\mu_{DP} - \mu_{NP}\|_1 = \left\| (\Lambda_0 + n\Lambda)^{-1}(\Lambda \sum_{i=1}^{n} \delta_i) \right\|_1$$
$$= \left\| (\frac{1}{n}\Lambda^{-1}\Lambda_0 + I)^{-1} \frac{1}{n} \sum_{i=1}^{n} \delta_i \right\|_1 \geq \frac{1}{2} \left\| \frac{1}{n} \sum_{i=1}^{n} \delta_i \right\|_1$$

for sufficiently large n. By the central limit theorem the distribution of $\frac{1}{n}\sum_{i=1}^{n} \delta_i$ converges to a Gaussian with non-zero variance. Hence μ_{DP} does not converge to μ_{NP} for large n and the method is not asymptotically consistent. □

1.3.1 Asymptotic efficiency

Theorem 5. *ϵ-differentially private estimate of the mean of a d-dimensional Gaussian variable x bounded by $\|x_i\|_1 \leq B$ in which the Laplace mechanism is used to perturb the sufficient statistics, is asymptotically efficiently private.*

Proof. In the proof of Theorem 3 we showed that

$$\|\mu_{DP} - \mu_{NP}\|_1 \leq \frac{c}{n}\|\delta\|_1,$$

where $\delta = (\delta_1, \ldots, \delta_d)^T \in \mathbb{R}^D$ with $\delta_j \sim$ Laplace $\left(0, \frac{2Bd}{\epsilon}\right)$.

Because δ_j is Laplace, $|\delta_j|$ is exponential with

$$|\delta_j| \sim \text{Exponential}\left(\frac{\epsilon}{2Bd}\right)$$

and

$$\|\delta\|_1 = \sum_{j=1}^{d} |\delta_j| \sim \text{Gamma}\left(d, \frac{\epsilon}{2Bd}\right).$$

Given $\alpha > 0$ we can choose $C > cF^{-1}(1-\alpha; d, \epsilon/(2Bd)$, where $F^{1}(x; a, b)$ is the inverse cumulative distribution function of the Gamma distribution with shape a and rate b, to ensure that

$$\Pr\left\{\|\mu_{DP} - \mu_{NP}\|_1 > \frac{C}{n}\right\} \leq \Pr\left\{\frac{1}{n}\|\delta\|_1 > \frac{C}{n}\right\} = \Pr\{\|\delta\|_1 > C\} < \alpha. \quad (7)$$

□

1.3.2 Convergence rate

We can further study the probability of making an error of at least a given magnitude as

$$\begin{aligned}\Pr\{\|\mu_{DP} - \mu_{NP}\|_1 \geq \phi\} &\leq \Pr\left\{\frac{c}{n}\|\delta\|_1 \geq \phi\right\} \\ &= \Pr\left\{\mathrm{Gamma}\left(d, \frac{n\epsilon}{2Bcd}\right) \geq \phi\right\} \\ &= 1 - F\left(\phi; d, \frac{n\epsilon}{2Bcd}\right) = 1 - \frac{\gamma(d, \frac{n\phi\epsilon}{2Bcd})}{\Gamma(d)}, \end{aligned} \tag{8}$$

where $F(x; a, b)$ is the cumulative distribution function of the Gamma distribution with shape a and rate b.

The formula in Eq. (8) unfortunately has no simple closed form expression. The result shows, however, that the n required to reach a certain level of performance is linear in B and $\frac{1}{\epsilon}$. The dependence on d is complicated, but it is in general super-linear as suggested by the mean of the gamma distribution in Eq. (8), $\frac{2Bd^2}{n\epsilon}$.

1.4 Example: Zhang et al., AAAI 2016, (arxiv:1512.06992)

In their paper Zhang et al. derive utility bounds for a number of mechanisms. The bounds are clearly insufficient to demonstrate the asymptotic efficiency of the corresponding methods. For Laplace mechanism applied to Bayesian network inference, their bound on excess KL-divergence as a function of the data set size n is

$$\mathcal{O}(mn \ln n)\left[1 - \exp\left(-\frac{n\epsilon}{2|\mathcal{I}|}\right)\right] + \sqrt{-\mathcal{O}(mn \ln n) \ln \delta}.$$

2 Differentially private linear regression

Let us next consider the linear regression model with fixed noise Λ,

$$\begin{aligned} y_i | x_i &\sim N(x_i^T \beta, \Lambda) \\ \beta &\sim N(\beta_0, \Lambda_0), \end{aligned}$$

with β as the unknown parameter and Λ and Λ_0 denoting the precision matrices of the corresponding distributions.

Let the observed data set be $\mathcal{D} = \{(x_i, y_i)\}_{i=1}^n$ with sufficient statistics $n\overline{xx} = \sum_{i=1}^n x_i x_i^T$ and $n\overline{xy} = \sum_{i=1}^n x_i y_i$.

The non-private posterior precision of β is

$$\Lambda_{NP} = \Lambda_0 + \Lambda n\overline{xx}$$

and the corresponding posterior mean is

$$\mu_{NP} = \Lambda_{NP}^{-1}(\Lambda n\overline{xy} + \Lambda_0\beta_0). \tag{9}$$

The corresponding private posterior precision is obtained by replacing $n\overline{xx}$ with the perturbed version $n\overline{xx}' = n\overline{xx} + \Delta$, where Δ follows the Laplace distribution according to the Laplace mechanism, yielding

$$\Lambda_{DP} = \Lambda_0 + \Lambda(n\overline{xx} + \Delta).$$

Similarly using $n\overline{xy}' = n\overline{xy} + \delta$ with δ following the Laplace mechanism we obtain

$$\mu_{DP} = \Lambda_{DP}^{-1}(\Lambda(n\overline{xy} + \delta) + \Lambda_0\beta_0). \tag{10}$$

As presented in Methods, a more robust alternative is to assign prior distributions to the precision parameters and then sample the posterior. This requires using the three sufficient statistics $n\overline{xx}$, $n\overline{xy}$, and $n\overline{yy}$ that are perturbed with suitable noise. The mechanism is presented in detail in Algorithm 1 and proven to guarantee differential privacy in Theorem 6. For theoretical analysis, we study the model with fixed precision parameters and an even privacy budget split between the two needed sufficient statistics. In Algorithm 1 and Theorem 6, this corresponds to setting $p_1 = p_2 = 0.5$ and leaving out the unnecessary term S_{yy}.

2.1 The detailed mechanism

The function PROJECT in Algorithm 1 projects the data points into a useful space and computes the sufficient statistics.

Theorem 6. *Algorithm* DIFFPRISS *in Algorithm 1 is ϵ-differentially private.*

Proof. (i) $S_{xx} = CC' + P$ is $p_1\epsilon$-differentially private.

S_{xx} is a symmetric $d \times d$ matrix with $\frac{d(d+1)}{2}$ degrees of freedom. After PROJECT $|C|_\infty \leq B_x$ and the sensitivity of each element $\Delta(S_{xx})_{ij} = \sup|c_i c_j - c_i' c_j'| \leq 2B_x^2$. Adding Laplace distributed noise to $(S_{xx})_{ij}$ with $b = \frac{d(d+1)B_x^2}{p_1\epsilon}$ yields an ϵ'-DP mechanism with $\epsilon' = \frac{2p_1\epsilon}{d(d+1)}$. Using basic composition [2] over the $\frac{d(d+1)}{2}$ independent dimensions shows that $S_{xx} = CC' + P$ is $p_1\epsilon$-differentially private.

(ii) CD is a $d \times 1$ vector where d is the cardinality of $\mathbb{I}$ and each element of CD is computed as follows:

$$\forall i \in \mathbb{I}, \;\; CD_i = \sum_{j=1}^{n} C_{ij} D_j, \tag{11}$$

where $|C_{ij}| \leq B_x$ and $|D_j| \leq B_y$, and thus the sensitivity of CD is $2dB_xB_y$. Thus, $S_{xy} = CD + Q$ is $p_2\epsilon$-differentially private.

Algorithm 1 Differentially private statistics release

Require: $p_1 + p_2 + p_3 = 1$

function DIFFPRISS(X, Y, ϵ, B_x, B_y, p_1, p_2, p_3)

  $n = |Y|$, $d = \dim(X)$

  $(C, D) = \text{PROJECT}(X, Y, B_x, B_y)$

  for $i \in \{1, \ldots, n\}$ **do**

    for $j \in \{i, \ldots, n\}$ **do**

      $P_{ij} = P_{ji} \sim \text{Laplace}\left(0, \frac{d(d+1)B_x^2}{p_1\epsilon}\right)$

    end for

  end for

  for $i \in \mathbb{I}$ **do**

    $Q_i \sim \text{Laplace}\left(0, \frac{2dB_xB_y}{p_2\epsilon}\right)$

  end for

  $R \sim \text{Laplace}\left(0, \frac{B_y^2}{p_3\epsilon}\right)$

  $S_{xx} = CC' + P$

  $S_{xy} = CD + Q$

  $S_{yy} = DD' + R$

  return S_{xx}, S_{xy}, S_{yy}

end function

function PROJECT(X, Y, B_x, B_y)

  for $j = 1$ to n **do**

    for $i = 1$ to d **do**

      $C_{ij} = \max(-B_x, \min(B_x, X_{ij}))$

    end for

    $D_j = \max(-B_y, \min(B_y, Y_j))$

  end for

  return C, D

end function

(iii) DD' is a scalar computed as

$$DD' = \sum_{j=1}^{n} D_j^2,$$

where $|D_j| \leq B_y$, and thus the sensitivity of DD' is B_y^2. Thus, $S_{yy} = DD' + R$ is $p_3\epsilon$-differentially private.

Therefore, releasing S_{xx}, S_{xy}, and S_{yy} together by DIFFPRISS is ϵ-differentially private. □

2.2 Asymptotic consistency and efficiency

Theorem 7. *Differentially private inference of the posterior mean of the weights of linear regression with Laplace mechanism to perturb the sufficient*

statistics is asymptotically consistent with respect to the posterior mean.

Proof. Using Eqs. (9)–(10) we can evaluate

$$\begin{aligned}
\|\mu_{DP} - \mu_{NP}\|_1 &= \left\|\Lambda_{DP}^{-1}(\Lambda(n\overline{xy} + \delta) + \Lambda_0\beta_0) - \Lambda_{NP}^{-1}(\Lambda n\overline{xy} + \Lambda_0\beta_0)\right\|_1 \\
&\leq \left\|\Lambda_{DP}^{-1}(\Lambda(n\overline{xy} + \delta) + \Lambda_0\beta_0) - \Lambda_{DP}^{-1}(\Lambda n\overline{xy} + \Lambda_0\beta_0)\right\|_1 \\
&\quad + \left\|\Lambda_{DP}^{-1}(\Lambda n\overline{xy} + \Lambda_0\beta_0) - \Lambda_{NP}^{-1}(\Lambda n\overline{xy} + \Lambda_0\beta_0)\right\|_1 \\
&= \left\|\Lambda_{DP}^{-1}\Lambda\delta\right\|_1 + \left\|(\Lambda_{DP}^{-1} - \Lambda_{NP}^{-1})(\Lambda n\overline{xy} + \Lambda_0\beta_0)\right\|_1 \\
&= \left\|(\Lambda_0 + \Lambda(n\overline{xx} + \Delta))^{-1}\Lambda\delta\right\|_1 \\
&\quad + \left\|\left[(\Lambda_0 + \Lambda(n\overline{xx} + \Delta))^{-1} \right.\right. \\
&\qquad \left.\left. - (\Lambda_0 + \Lambda(n\overline{xx}))^{-1}\right](\Lambda n\overline{xy} + \Lambda_0\beta_0)\right\|_1 \\
&= \left\|(\Lambda_0 + \Lambda(n\overline{xx} + \Delta))^{-1}\Lambda\delta\right\|_1 \\
&\quad + \left\|\left[\left(\frac{1}{n}\Lambda_0 + \Lambda\left(\overline{xx} + \frac{1}{n}\Delta\right)\right)^{-1} \right.\right. \\
&\qquad \left.\left. - \left(\frac{1}{n}\Lambda_0 + \Lambda\overline{xx}\right)^{-1}\right]\left(\Lambda\overline{xy} + \frac{1}{n}\Lambda_0\beta_0\right)\right\|_1 .
\end{aligned}$$

Assuming $\overline{xx} > 0$, the first term clearly approaches 0 as $n \to \infty$. For the second term, as $n \to \infty$, $(\frac{1}{n}\Lambda_0 + \Lambda(\overline{xx} + \frac{1}{n}\Delta))^{-1} \to (\frac{1}{n}\Lambda_0 + \Lambda\overline{xx})^{-1}$ and as $(\Lambda\overline{xy} + \frac{1}{n}\Lambda_0\beta_0)$ is bounded, the second term also approaches 0 as $n \to \infty$. This shows that μ_{DP} converges in probability to μ_{NP}. □

Theorem 8. *ϵ-differentially private inference of the posterior mean of the weights of linear regression with the Laplace mechanism of Algorithm 1 to perturb the sufficient statistics is asymptotically efficiently private.*

Proof. From the proof of Theorem 7 we have

$$\begin{aligned}
&\|\mu_{DP} - \mu_{NP}\|_1 \leq \left\|(\Lambda_0 + \Lambda(n\overline{xx} + \Delta))^{-1}\Lambda\delta\right\|_1 \\
&+ \left\|\left[\left(\frac{1}{n}\Lambda_0 + \Lambda\left(\overline{xx} + \frac{1}{n}\Delta\right)\right)^{-1} - \left(\frac{1}{n}\Lambda_0 + \Lambda\overline{xx}\right)^{-1}\right]\left(\Lambda\overline{xy} + \frac{1}{n}\Lambda_0\beta_0\right)\right\|_1 .
\end{aligned} \tag{12}$$

The first term can be bounded easily as

$$\begin{aligned}
\left\|(\Lambda_0 + \Lambda(n\overline{xx} + \Delta))^{-1}\Lambda\delta\right\|_1 &= \left\|(\Lambda^{-1}\Lambda_0 + \Delta + n\overline{xx})^{-1}\delta\right\|_1 \\
&\leq \left\|(\Lambda^{-1}\Lambda_0 + \Delta + n\overline{xx})^{-1}\right\|_1 \|\delta\|_1 \\
&\leq \frac{c_1}{n}\left\|(\overline{xx})^{-1}\right\|_1 \|\delta\|_1
\end{aligned} \tag{13}$$

where $c_1 > 1$. The bound is valid for any $c_1 > 1$ as n gets large enough.

Similarly as in the proof of Theorem 5,

$$\|\delta\|_1 \sim \text{Gamma}\left(d, \frac{\epsilon}{4dB_x B_y}\right). \tag{14}$$

Given $\alpha > 0$ we can choose similarly as in the proof of Theorem 5

$$C_1 > c_1 F^{-1}(1-\alpha/2; d, \epsilon/(4dB_x B_y)) \left\|(\overline{xx})^{-1}\right\|_1,$$

where $F^{-1}(x; \alpha, \beta)$ is the inverse distribution function of the Gamma distribution with shape α and rate β, to ensure that

$$\Pr\left\{\|(\Lambda_0 + \Lambda(n\overline{xx} + \Delta))^{-1}\Lambda\delta\|_1 > \frac{C_1}{n}\right\} < \frac{\alpha}{2}. \tag{15}$$

The second term can be bounded as

$$\begin{aligned}
&\left\|\left[\left(\frac{1}{n}\Lambda_0 + \Lambda\left(\overline{xx} + \frac{1}{n}\Delta\right)\right)^{-1} - \left(\frac{1}{n}\Lambda_0 + \Lambda\overline{xx}\right)^{-1}\right]\left(\Lambda\overline{xy} + \frac{1}{n}\Lambda_0\beta_0\right)\right\|_1 \\
&= \left\|\left[\left(\frac{1}{n}\Lambda^{-1}\Lambda_0 + \overline{xx} + \frac{1}{n}\Delta\right)^{-1} - \left(\frac{1}{n}\Lambda^{-1}\Lambda_0 + \overline{xx}\right)^{-1}\right]\left(\overline{xy} + \frac{1}{n}\Lambda^{-1}\Lambda_0\beta_0\right)\right\|_1 \\
&= \frac{1}{n}\left\|\left(\frac{1}{n}\Lambda^{-1}\Lambda_0 + \overline{xx} + \frac{1}{n}\Delta\right)^{-1}\Delta\left(\frac{1}{n}\Lambda^{-1}\Lambda_0 + \overline{xx}\right)^{-1}\left(\overline{xy} + \frac{1}{n}\Lambda^{-1}\Lambda_0\beta_0\right)\right\|_1 \\
&\leq \frac{1}{n}\left\|\left(\frac{1}{n}\Lambda^{-1}\Lambda_0 + \overline{xx} + \frac{1}{n}\Delta\right)^{-1}\Delta\left(\frac{1}{n}\Lambda^{-1}\Lambda_0 + \overline{xx}\right)^{-1}\right\|_1 \left\|\overline{xy} + \frac{1}{n}\Lambda^{-1}\Lambda_0\beta_0\right\|_1 \\
&\leq \frac{1}{n}\left\|\left(\frac{1}{n}\Lambda^{-1}\Lambda_0 + \overline{xx} + \frac{1}{n}\Delta\right)^{-1}\right\|_1 \|\Delta\|_1 \\
&\qquad \left\|\left(\frac{1}{n}\Lambda^{-1}\Lambda_0 + \overline{xx}\right)^{-1}\right\|_1 \left\|\overline{xy} + \frac{1}{n}\Lambda^{-1}\Lambda_0\beta_0\right\|_1 \\
&\leq \frac{c_2}{n}\left\|(\overline{xx})^{-1}\right\|_1 \|\Delta\|_1 \left\|(\overline{xx})^{-1}\right\|_1 \|\overline{xy}\|_1 =: \frac{c_2}{n}\mathcal{B}_2,
\end{aligned}$$

where similarly as in Eq. (13), the bound is valid for any $c_2 > 1$ as n gets large enough. Here $\|\Delta\|_1$ is the l_1-norm of the matrix Δ that whose elements follow the Laplace distribution $\Delta_{ij} \sim \text{Laplace}(0, \frac{2d(d+1)B_x^2}{\epsilon})$. We can bound it as

$$\|\Delta\|_1 = \max_i \|\Delta_{:i}\|_1,$$

where $\Delta_{:i}$ are the row vectors of Δ and the latter is the vector ℓ_1-norm. Similarly as in Eq. (14) we have

$$\|\delta\|_1 \sim \text{Gamma}\left(d, \frac{\epsilon}{2d(d+1)B_x^2}\right) \tag{16}$$

and as above given $\alpha > 0$ we can choose

$$C_2 > c_2 F^{-1}(1-\alpha/2; d, \epsilon/(2d(d+1)B_x^2)) \left\|(\overline{xx})^{-1}\right\|_1^2 \|\overline{xy}\|_1 ,$$

where $F^{-1}(x;\alpha,\beta)$ is the inverse distribution function of the Gamma distribution to ensure that

$$\Pr\left\{\mathcal{B}_2 > \frac{C_2}{n}\right\} < \frac{\alpha}{2}. \tag{17}$$

Combining Eqs. (15) and (17) shows that

$$\Pr\left\{\|\mu_{DP} - \mu_{NP}\|_1 > \frac{C_1 + C_2}{n}\right\} < \alpha. \tag{18}$$

□

2.3 Convergence rate

Using Chebysev's inequality together with Eq. (14) we can show that with high probability

$$\|\delta\|_1 = \mathcal{O}\left(\frac{d^2 B_x B_y}{\epsilon}\right)$$

and thus

$$\left\|(\Lambda_0 + \Lambda(n\overline{xx} + \Delta))^{-1}\Lambda\delta\right\|_1 = \mathcal{O}\left(\frac{d^2 B_x B_y \left\|(\overline{xx})^{-1}\right\|_1}{n\epsilon}\right). \tag{19}$$

Similarly for the second term we obtain

$$\mathcal{B}_2 = \mathcal{O}\left(\frac{d^3 B_x^2 \left\|(\overline{xx})^{-1}\right\|_1^2 \|\overline{xy}\|_1}{\epsilon}\right). \tag{20}$$

Combining Eqs. (12)–(20) yields

$$\|\mu_{DP} - \mu_{NP}\|_1 = \mathcal{O}\left(\frac{d^2 B_x B_y \|\overline{xx}^{-1}\|_1 + d^3 B_x^2 \left\|(\overline{xx})^{-1}\right\|_1^2 \|\overline{xy}\|_1}{n\epsilon}\right)$$

with high probability.

References

[1] P. Diaconis and D. Ylvisaker. Conjugate priors for exponential families. *Ann. Stat.*, 7(2):269–281, Mar 1979.

[2] C. Dwork and A. Roth. The algorithmic foundations of differential privacy. *Found. Trends Theor. Comput. Sci.*, 9(3-4):211–407, Aug. 2014.

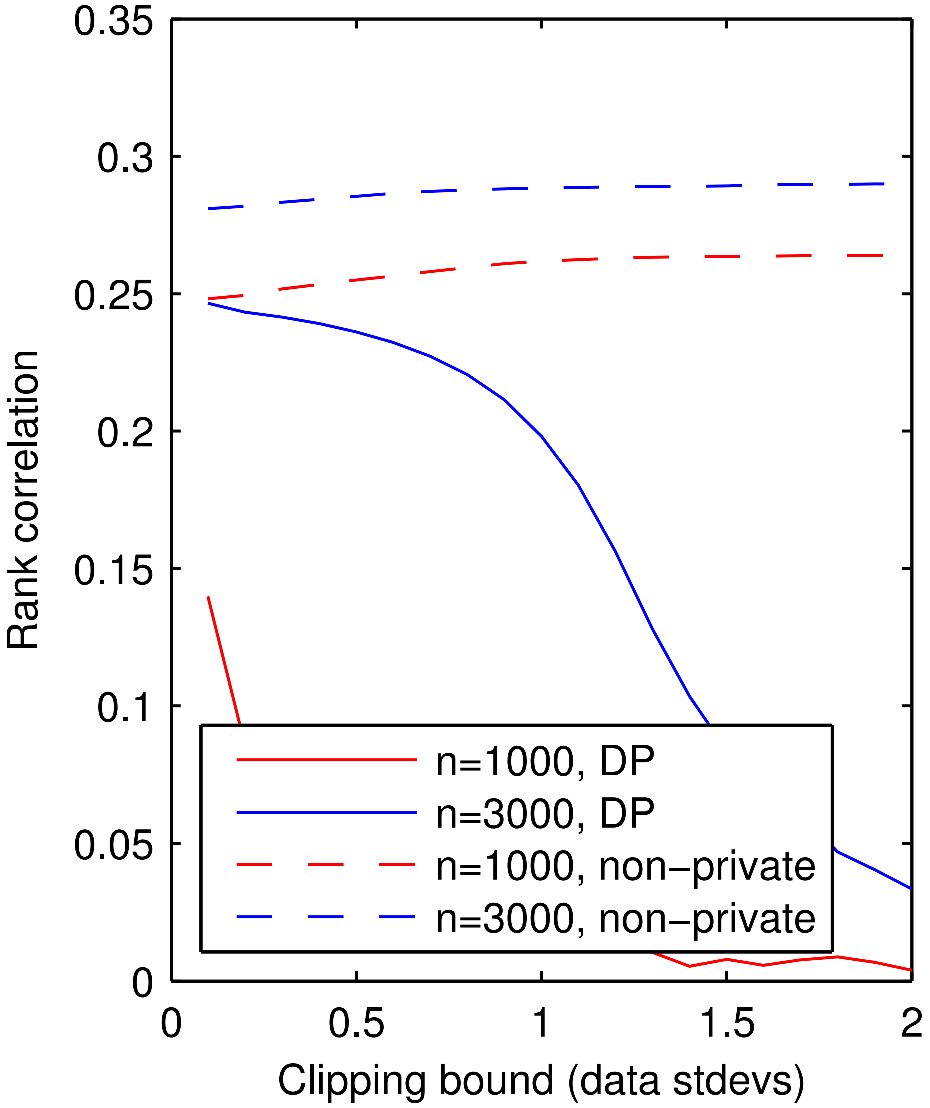

Figure S1: This is a complement to Figure 4 in the main text. The figure illustrates the effect of projecting the outliers to within the bounds in linear regression, for different sample sizes n with 15-dimensional synthetic data, evaluated by Spearman's rank correlation between the predicted and true values (higher values are better), both for DP (solid lines) and non-private regression (dashed lines). The lines show a minor decrease in accuracy of the non-private algorithm as the projection threshold becomes increasingly tight. This minor decrease is eclipsed by a dramatic increase in the accuracy of the DP algorithm.

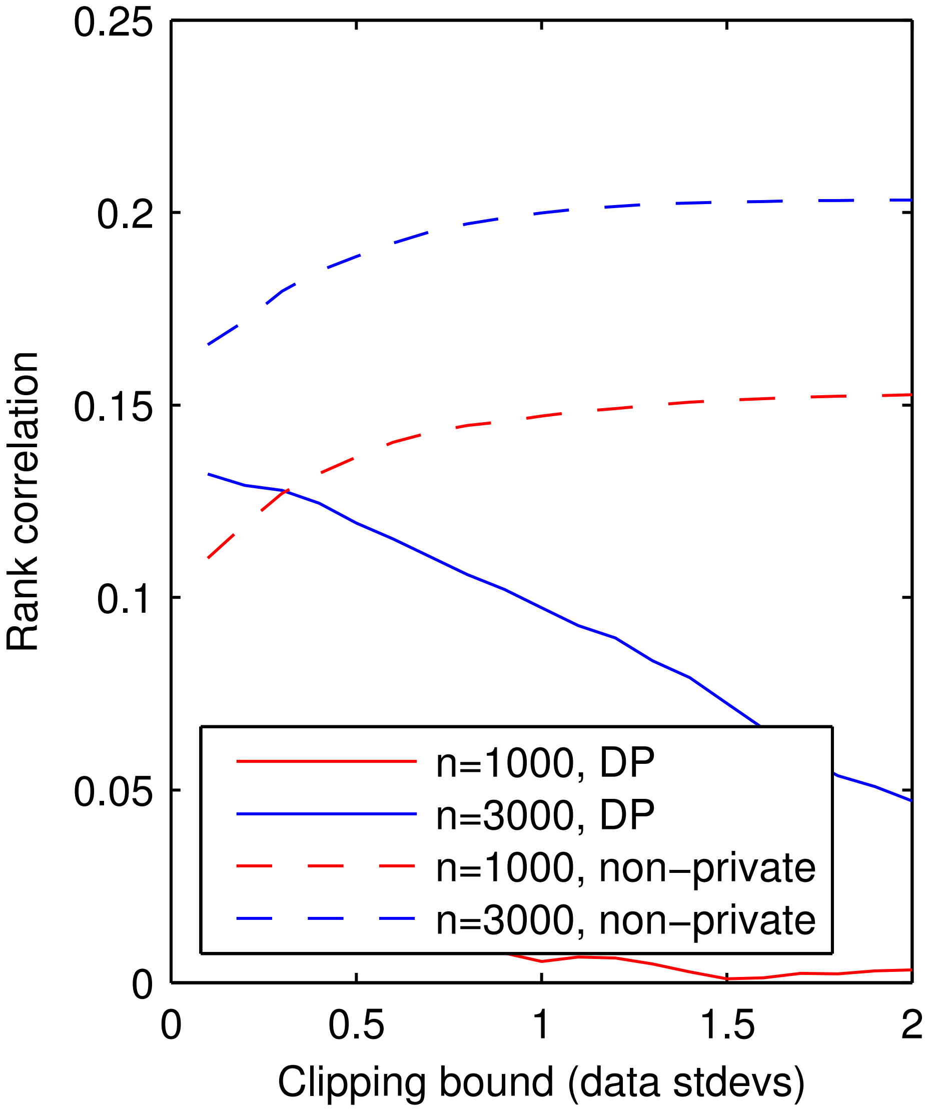

Figure S2: This is a complement to Figure 4 in the main text. The figure illustrates the effect of projecting the outliers to within the bounds in linear regression, for different sample sizes n with 10-dimensional synthetic data sampled from Student's-t distribution with degrees of freedom as 1 , evaluated by Spearman's rank correlation between the predicted and true values (higher values are better), both for DP (solid lines) and non-private regression (dashed lines). The lines show a minor decrease in accuracy of the non-private algorithm as the projection threshold becomes increasingly tight. This minor decrease is eclipsed by a dramatic increase in the accuracy of the DP algorithm.

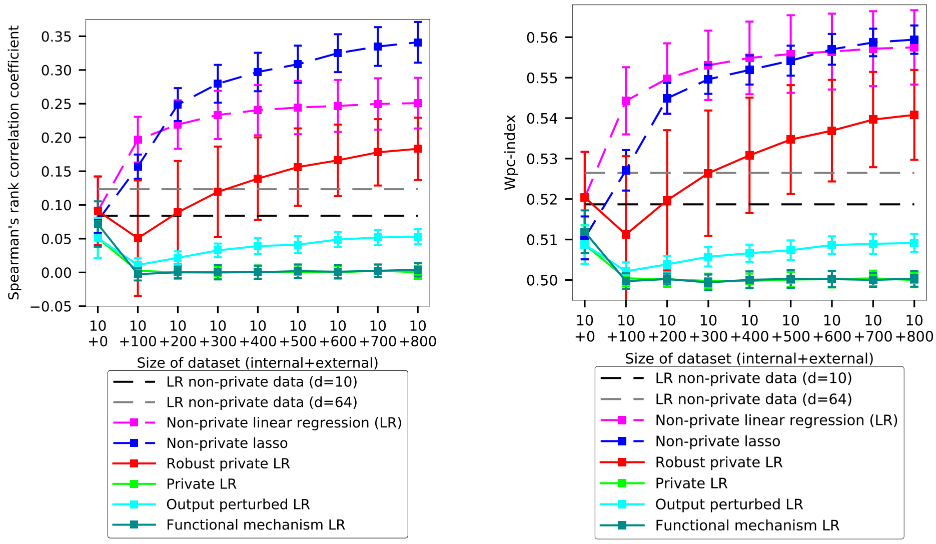

Figure S3: This is a complement to Figure 5 in the main text with more stringent privacy. Here we show Spearman's rank correlation coefficients (ρ, left) and wpc-index (right) between the measured ranking of the cell lines and the ranking predicted by the models using $\epsilon = 1$. The baselines (horizontal dashed lines) are learned on 10 non-private data points; the private algorithms additionally have privacy-protected data (x-axis). The non-private algorithm (LR) has the same amount of additional non-privacy-protected data. All methods use 10-dimensional data except the gray baseline showing the best performance with 10 non-private 64-dimensional data points. The results are averaged over all drugs and 50-fold Monte Carlo cross-validation; error bars denote standard deviation over 50 Monte Carlo repeats. The result shows that more data are needed for good prediction performance under more stringent privacy.

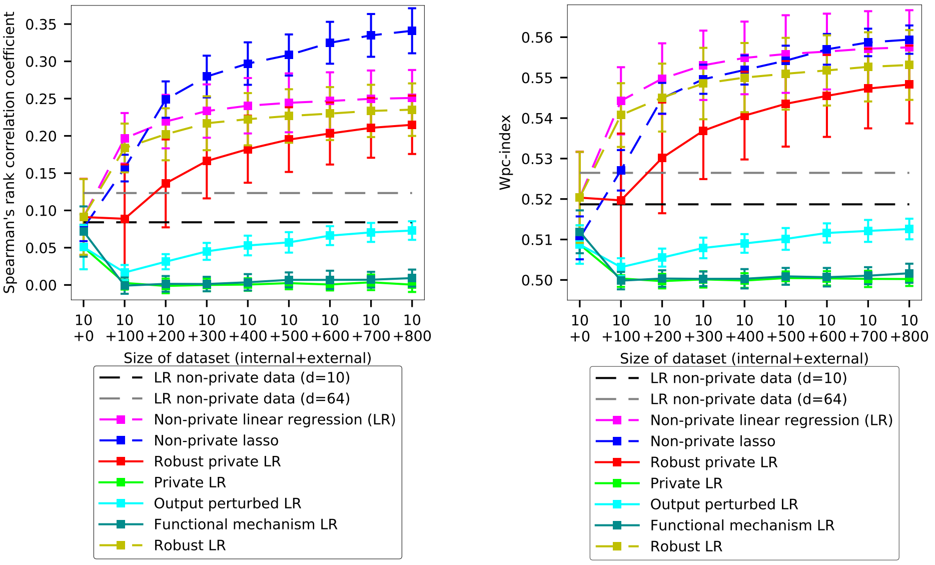

Figure S4: This is a complement to Figure 5 in the main text with inclusion of robust LR. Here we show Spearman's rank correlation coefficients (ρ, left) and wpc-index (right) between the measured ranking of the cell lines and the ranking predicted by the models using $\epsilon = 2$. The baselines (horizontal dashed lines) are learned on 10 non-private data points; the private algorithms additionally have privacy-protected data (x-axis). The non-private algorithm (LR) has the same amount of additional non-privacy-protected data. All methods use 10-dimensional data except the gray baseline showing the best performance with 10 non-private 64-dimensional data points. The results are averaged over all drugs and 50-fold Monte Carlo cross-validation; error bars denote standard deviation over 50 Monte Carlo repeats. The result shows that more data are needed for good prediction performance under more stringent privacy.
